# The Coming Elections

**Published:** 1919-01-25

**Authors:** 


### THE COLLEGE OF NURSING.

### The Coming Elections.

For whom do you intend to vote, and why? Uninterested in the claims of any individual, I put the question on general grounds, because it is of pressing and far-reaching importance. The building of the College, spiritual and material, depends on the nurse, and not even in the smallest degree on its Articles of Association or on the fact that it comes within the legal definition of a company limited by guarantee. All this is controversial trickery, deliberately concocted by the enemies of the College and calculated to deceive. If the nurse's voice were silenced by rule and regulation, I for one would be on the side of the enemy. I should endeavour, to the best of my ability, to pull down the walls of any fabric designed to forge for her further chains of slavery. The College, according to its splendidly conceived constitution, leaves it absolutely at the discretion of the nurse to elect whomsoever she will to direct her destiny. Whenever vacancies arise the nurse can nominate, and she can likewise vote. The task may be troublesome, and I can conceive of situations where it may require some courage and initiative. But every achievement, individual or collective, involves both trouble and serious thought.

### A Conversation in Ireland.

I happened the other day to be present at a conversation which may have a bearing on these remarks. It took place in Ireland between two men, and, although it occupied only a few moments, it gave me much cause for reflection. One was a big, virile man of the world, and the other, his friend of long standing, a sort of *malade imaginaire*, the victim of a frail body and many nerves. "I have just come out of a hospital," remarked the delicate one. "You know it," he said, and he gave the name of a high-class private nursing home. "And how did you fare?" inquired the other. "Splendidly," he said. "A little dull and lonesome at times, but good nursing." "The matron," he went on, "is ideal. She knows her work thoroughly, and does it. There are no mistakes." And then, after a moment, he added as an afterthought, "Of course, she is a dreadful tyrant to the nurses. They are in mortal terror. But from the patient's point of view, from my point of view, this is good. It is necessary. Nursing is a dog's life!" And so the conversation drifted on to other matters. I said not a word, but I thought much. The picture was vivid. The tyrant is necessary because it is a dog's life!

### "A High-class Private Nurses' Home."

I happen to know this particular establishment, and I happen to be aware that the lady concerned is a member of the College of Nursing Executive. I wonder whom she represents, or what part she is likely to play in advocating economic reform for the women who regard her as a "dreadful tyrant"? Obviously the financial success of the home depends on the wages and the working hours of the nurse. Pray do not accuse me of prejudice. There are judges on the King's Bench who are severe in the administration of laws which they would wish to see repealed, and this particular College Senator may be a very Bolshevist in her executive capacity. But, on the other hand, she may not, and instead she may be a mountain of opposition to economic reform. The working nurse can form her own conclusions.

### For Whom will You Vote?

I repeat my inquiry. For whom do you intend to vote, and why? I admit that courage and initiative may be necessary in order to ensure that the Executive shall conform to the ideals of the people whom they are supposed to represent. Straightforward and uncompromising questions may have to be put. It is vain to deny that there are still matrons who regard the voice of the nurse as something that ought never to be heard above a whisper, and who believe that she is utterly unfitted for even an elementary form of self-determination. If this class of person were ever to dominate the College Executive they would involve it in general ruin. I do not suggest that such will ever be the case, but I venture emphatically to point out that the nurse cannot afford to take anything for granted. The coming election is going to be a very crucial affair, and if the nurse takes no interest in the proceedings, and makes no effort either to find out the views and intentions of candidates who are nominated by others or to make her own nominations she will find it difficult to complain at a later stage if she is disappointed with the progress.

### Wanted: Candidates' Manifestoes.

Moreover, candidates for election should issue without request their individual manifestoes. There is a feeling in the nursing world that we know too little of the mind of the Executive, collectively and individually. What they have achieved, what they have attempted to achieve, what they hope to achieve—all these things ought to be made public regularly and frequently. Those who attend, and the number of attendances ought to be on record, so that nurses may realise how their representatives guard their interests and fulfil the trust. Build up your College, O ye nurses! Build it of stone, and make of it an edifice that will endure the shock of storm and time. Light it with the lamp of truth, so that the wayfaring man may see and admire and offer his tribute. The elections that are at hand present a golden opportunity. It is for every one of the 11,000 electors to see to it that each representative for whom she votes is pledged to a policy of immediate reform.

IERNE.

